# Self-organization of cellulose-producing microbial communities during biofilm spreading

**DOI:** 10.1039/d5sm00720h

**Published:** 2025-10-31

**Authors:** Julie M. Laurent, Anton Kan, Mathias Steinacher, André R. Studart

**Affiliations:** a Complex Materials, Department of Materials, ETH Zürich 8093 Zürich Switzerland andre.studart@mat.ethz.ch

## Abstract

Matrix-secreting microorganisms form self-organizing biofilms that provide protection and mechanical robustness to the embedded microbial communities. Biofilms made by cellulose-producing bacteria from *Komagataeibacter* species are widely used for food and bio-manufacturing, but their self-organization in mixed microbial communities has not yet been reported. Here, we investigate the self-organization and spreading of biofilm communities comprising distinct cellulose-producing variants of *K. sucrofermentans*. Using fluorescently labeled strains grown on solid culture medium, mixed pairs of variants produced striking spatial patterns, with distinct strains dominating the inner and outer regions of the biofilm. The experiments reveal that pattern formation and the enrichment of one strain in the microbial biofilms are affected by the growth rate, cellulose-production rate, and expansion rate of the constituent bacterial strains. Friction between the cellulose-producing bacteria and the underlying substrate was found to be an important phenotype governing cell segregation in the microbial communities, while cell dominance within the biofilm was linked to the cellulose-producing ability of each strain. Understanding the effect of these traits on the cell composition and structure of microbial communities provides new control parameters to tune the formation of biofilms made by mixed cellulose-producing variants.

## Abstract 2

Cellulose-producing bacteria grown pairwise on solid substrates form exquisite spatial patterns during biofilm spreading. We offer design principles to control and tune cellulose biofilm structure for potential biofabrication and food applications.

## Graphical abstract

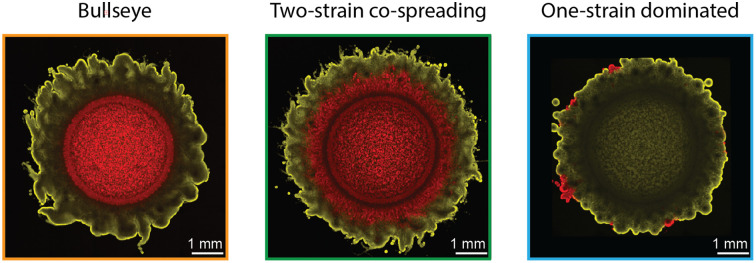

## Introduction

Microbial communities play major roles in human health, environmental processes, and biotechnological applications. They are crucial for the proper functioning of the human gastrointestinal tract,^1–3^ essential to global biogeochemical cycling,^4–6^ and can be used in wastewater treatment,^7,8^ or as biofertilizers and biopesticides in agriculture.^9,10^ Living in dense, often matrix-embedded communities provides microorganisms with increased resistance to environmental stressors.^11,12^ While robust biofilms can be beneficial, they also pose challenges in unwanted settings, such as antibiotic-resistant infections^13^ and industrial biofouling.^14–16^ These effects and the widespread occurrence of biofilms have driven efforts to understand their formation, stability, and the underlying cell–cell interactions. A deeper understanding of biofilm organization and structural heterogeneity could enable the development of strategies to combat biofilm-mediated diseases, control biofilm formation, and engineer functional synthetic communities. Notably, microbes in communities often self-organize into distinct spatial patterns, influencing their function and resilience.

The self-organization of bacterial communities generates spatial patterns during biofilm spreading on nutrient-rich solid surfaces. These spatial patterns emerge from the interplay between cell cooperation and competition in response to gradients imposed by the environment.^17–20^ In cooperative scenarios, the different species or strains intermix to enhance communal benefits. By contrast, competition for space and nutrients leads to spatial segregation of species during spreading. In the particular case of non-motile matrix-forming bacteria, several passive sliding mechanisms may contribute to biofilm spreading, including the formation of osmotic pressure gradients, the release of surfactants, and the generation of mechanical forces through cell proliferation.^21–26^ The production of extracellular matrix not only enables biofilm spreading toward nutrient-rich environments but can also facilitate microbial movement through directed motility. While the spreading behavior of such matrix-forming microorganisms has been thoroughly studied, the role of genetic variation and phenotypic traits in these spatial dynamics remains an open research topic.

Cellulose-producing bacteria exhibit a unique mode of motility driven by the extrusion of crystalline polymer fibers.^27–29^*Komagataeibacter* species, for instance, are Gram-negative, rod-shaped bacteria that secrete cellulose at the air-medium interface under static growth conditions, forming a gel-like pellicle that can be easily harvested. Given the industrial interest and broad applications of bacterial cellulose, efforts have been made to engineer strains for enhanced fiber production either using synthetic biology or directed evolution approaches. Overproducing variants have been generated by directly overexpressing the cellulose biosynthesis machinery,^30,31^ increasing the availability of cellulose precursors,^32–34^ or blocking competing metabolic pathways.^35–38^ A recent study using directed evolution also identified a mutation in a protease subunit that contributes to enhanced cellulose production.^39^ These findings raise fundamental questions about how genetic differences and altered cellulose production in these microorganisms may influence the spatial organization of microbial communities formed by distinct variants.

Here, we study the self-organization of cellulose-producing microbial communities using genetically and phenotypically distinct *K. sucrofermentans* variants. The genetic difference in the bacteria lies in the presence of mutations in genes encoding a protease complex, which was found to contribute to the amount of cellulose produced by the microorganism. Combining two genetically distinct bacteria with native and control microorganisms provided ample parameter space to investigate the self-organization of cellulose-producing microbial communities during biofilm spreading on a solid substrate. For this, we experimentally investigate the spreading of cellulose-producing bacteria in single and mixed microbial biofilms. Finally, we analyze the effect of distinct phenotypic traits on the cell segregation and composition of mixed biofilms to shed light on the physical processes that govern the self-organization of cellulose-forming bacteria into unique spatial patterns.

## Results and discussion

**Fig. 1:**
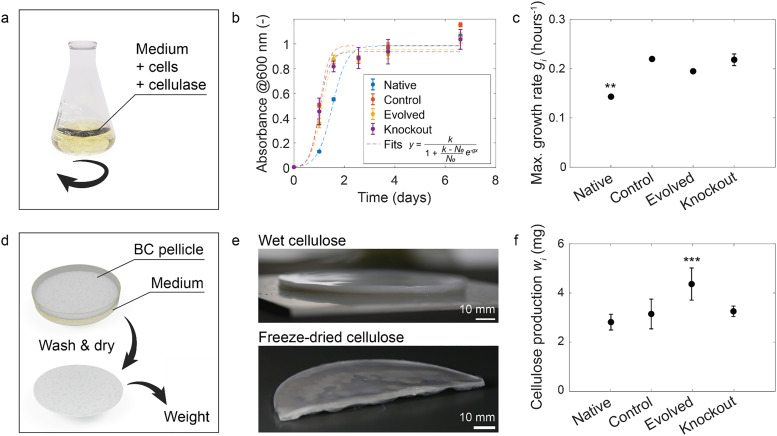
Properties of individual cellulose-producing bacterial strains. (a) Cells were grown in liquid medium containing 2 vol% cellulase (200 rpm, 28 °C, 80% RH). (b) Absorbance-based growth curves obtained for all bacterial strains and fitted using the logistic growth model (dashed lines). (c) Maximum growth rate (*g*_i_) extracted from the model for all bacterial strains (*n* = 3). (d) Bacterial cellulose, grown for 12 days in 5 mL liquid medium (static, 28 °C, 80% RH), was washed, dried, and weighed. (e) Representative images of bacterial cellulose pellicles formed by the Evolved bacteria in larger cultures (2× medium). (f) Amount of cellulose produced (*w*_i_) by the different bacterial strains (*n* = 9). Error bars represent the standard deviations. Statistics: **P < 0.01, ***P < 0.001. i = {Native, Control, Evolved, Knockout}.

Four variants of *K. sucrofermentans* were investigated in terms of self-organization in spreading microbial communities: Native, Control, Evolved, and Knockout strains. The Control and Native strains are genetically identical and differ only in the chemical environments to which they were exposed before testing. The Native strain is directly taken from available cultures, whereas the Control variant corresponds to the Native microorganism after exposure to 0.9% NaCl solution for 1 hour, which we found to directly impact its growth rate and cellulose production (Fig. 1).^39^ In contrast to these genetically unmodified variants, the Evolved and Knockout strains display mutations in genes that encode the interacting ClpA and ClpS sub-units of the protease complex ClpAPS, respectively.^39^ The mutation in the Evolved strain is a 12-base-pair deletion in the *clpA* gene, which leads to a stable phenotype over at least 5 passages.^39^ The genetic change in the Knockout variant (ΔclpS::cat) consists of a deletion in the start codon of the *clpS* gene. We note that both the Control and the Evolved strains were exposed to 0.9% NaCl, but only the Evolved microorganisms are genetically modified.^39^ Altogether, these genetic and environmental factors were found to significantly affect the phenotypic traits of the four variants.

The most distinct phenotypic traits of the investigated bacteria are their growth behavior and cellulose-forming ability in culture medium. To compare these phenotypic traits, we first measured the cell growth and cellulose production ability of the four strains. Growth curves of individual bacterial strains were obtained from absorbance measurements of cells in shaking liquid medium containing 2 vol% cellulase (Fig. 1a and b). The addition of cellulase to the medium enabled the quantification of cell proliferation alone, with limited influence from cellulose fibers. The growth experiments reveal that the Evolved, Knockout, and Control strains proliferate significantly faster than the Native bacteria (Fig. 1c). To quantify this behavior, we calculated the maximum growth rate (*g*_i_) of individual strains by fitting the logistic growth model to the absorbance data. The Evolved, Knockout, and Control bacteria showed a mean growth rate in the range 0.19–0.22 hours^−1^ (∓0.01), which was found to be statistically 36–57% higher than the proliferation speed estimated for the Native strain (0.14 ∓ 0.001 hours^−1^).

In addition to cell growth, the individual bacterial strains were also characterized in terms of cellulose production (*w*_i_) during incubation in static culture medium. Incubation for 12 days led to the formation of thick cellulose pellicles at the air–water interface of the liquid culture bath (Fig. 1d and e). After incubation, wet pellicles formed by the different bacteria were washed with NaOH to remove cells, dried, and weighed with a laboratory scale. The results showed that the Evolved strain produced significantly more cellulose than the other strains (Fig. 1f). With a mean dry weight of 4.36 mg (∓0.653 mg), pellicles made by the Evolved variant in 5 mL of liquid culture were 34–55% heavier than samples formed by the other investigated microorganisms.

**Fig. 2:**
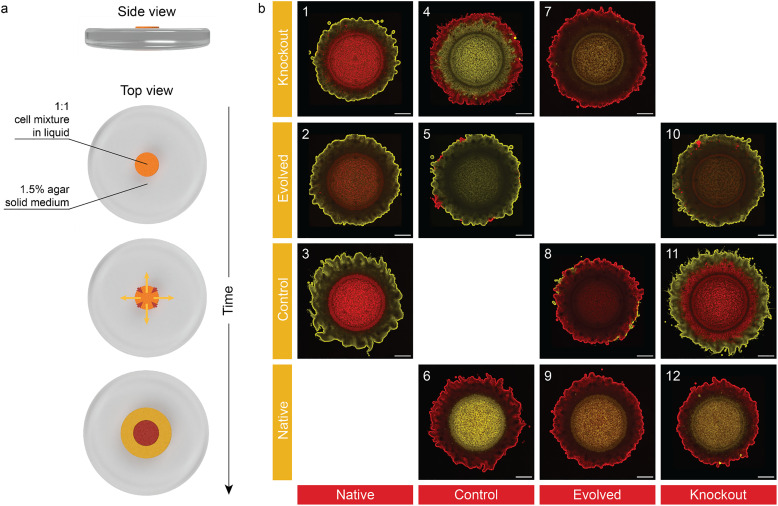
Cellulose-producing microbial communities self-organize when grown on agar. (a) RFP- and YFP- expressing bacterial strains were mixed in 1 : 1 ratio before deposition on top of 1.5% agar solid medium. The bacteria from different strain pairs self-organized over time in the formed biofilm. (b) Self-organized patterns observed after 11 days for the different bacterial strain pairs (bullseye: 1, 2, 3, 6, 9, 12; one-strain dominated: 5, 7, 8, 10; two-strain co-spreading: 4, 11). Scale bars: 1 mm.

The different phenotypic traits of the four investigated bacteria strongly affect the self-organization of microbial communities formed by these cellulose-producing microorganisms. To illustrate this, we performed spreading experiments using communities containing equal proportions of two distinct bacteria in the initial inoculum (Fig. 2a). In this experiment, the inoculum was deposited on a nutrient-rich 1.5% agar substrate and imaged after 11 days in a confocal microscope to study the self-organization of the growing microbial community. To differentiate the two cell types in communities, the bacteria variants were genetically engineered to chromosomally express either yellow (YFP) or red (RFP) fluorescent proteins (see Materials and methods). The cell density of each variant in the final biofilm was quantified by summing *z*-stack images obtained from red and yellow fluorescence channels.

Confocal images show that the spreading bacterial communities self-organize into a variety of exquisite microbial patterns, depending on the pair of strains present in the inoculum (Fig. 2b). The different spatial patterns can be qualitatively grouped into 3 main categories: (1) bullseye, (2) one-strain dominated, and (3) two-strain co-spreading. In the bullseye pattern (pairs 1, 2, 3, 6, 9, and 12 in Fig. 2b), one strain remains within the area of the original inoculum in the inner part of the biofilm (core), while the other dominates the outer part of the film (edge). In the one-strain dominated pattern (pairs 5, 7, 8, and 10), the biofilm contains mostly one bacterial strain, with the less-abundant variant occasionally occupying small sites at the edge of the biofilm. Finally, the two-strain co-spreading pattern (pairs 4 and 11) displays a segregated pattern but with the core strain also spreading away from the original inoculum. The formation of such self-organized patterns is surprising in view of the small genotypic and phenotypic differences between the investigated strains. Extensive cell segregation is observed even among genetically identical variants, as shown by the bullseye pattern formed by films containing the Native and Control strains (Fig. 2b).

**Fig. 3:**
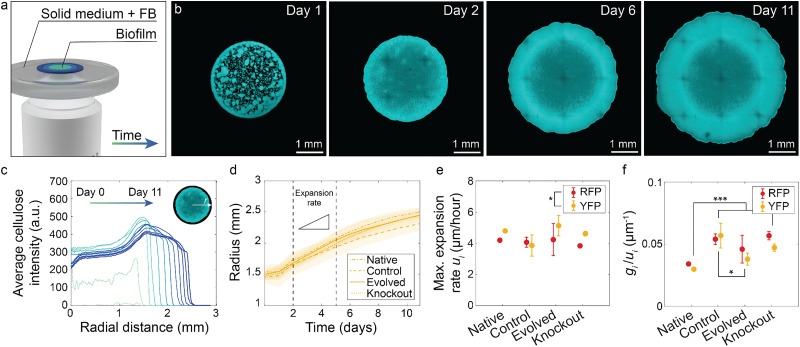
Expansion rate of biofilms produced by individual bacterial strains. (a) Cell suspensions were deposited on top of 1.5% agar solid medium containing fluorescent brightener (FB), a cellulose-binding dye. Biofilms expand radially over time. (b) Representative confocal images of cellulose biofilms at different time points for the YFP Native strain. Images from all strains are shown in Fig. S1. (c) Representative azimuthal average of the cellulose intensity from confocal images at different time points (YFP Native strain). (d) Radius of cellulose biofilms over time extracted from the integrated cellulose-fluorescence signal for all YFP-expressing strains. The intensity profiles obtained for RFP-expressing strains are displayed in Fig. S3. Shaded areas around the curves represent the standard deviations. (e) Maximum radial expansion rate *u*_i_ calculated between day 2 and day 5 for all strains. Data for RFP and YFP strains are shown in red and yellow, respectively. (f) Ratio *g*_i_/*u*_i_ calculated from expansion and growth experiments for all strains (RFP strains in red and YFP strains in yellow). Error bars represent standard deviations. Statistics: *P < 0.05, ***P < 0.001, *n*_biofilm_ = 3. i = {Native_RFP_, Native_YFP_, Control_RFP_, Control_YFP_, Evolved_RFP_, Evolved_YFP_, Knockout_RFP_, Knockout_YFP_}.

To better understand the origin of the spatial patterns in the self-organizing microbial communities, we investigated the spreading behavior of microbial biofilms containing one single bacterial strain (Fig. 3). In this experiment, individual bacterial strains expressing either RFP or YFP were inoculated on top of solid agar medium containing a cellulose-binding fluorescent dye. Because of the low RFP and YFP signals of the initial inoculum, the fluorescent dye was used to image the cellulose-producing biofilm during spreading under a confocal microscope for 11 days (Fig. 3a, b and Fig. S1). The fluorescence signal acquired from the images was azimuthally integrated to obtain the radial intensity profiles of the cellulose biofilms as a function of time (Fig. 3c). Complementary experiments have shown that the addition of the cellulose-binding dye in the agar did not affect the biofilm spreading (Fig. S2).

The radial intensity profiles show a strong increase in fluorescence during the first two days of the experiment, resulting from the production of cellulose within the initial inoculum until full coverage of the area (Fig. 3c). From the second day, the outer edge of the fluorescence profile progressively advances, indicating radial expansion of the biofilm. The diameter of the microbial colony grows continuously over time with a maximum expansion rate between days 2 and 5 (Fig. 3d). Experiments with different biofilms revealed that the maximum expansion rate showed no statistically significant differences between biofilms made from the distinct strains (Fig. 3e).

Earlier research has shown that the radial expansion of non-motile, matrix-producing microorganisms can occur through several passive sliding mechanisms, including osmotic gradients, surfactant synthesis, and mechanical forces generated during cell division.^21–26^ By consuming water and nutrients while producing extracellular matrix and secreting metabolites, cells increase the local osmotic pressure in the biofilm, providing a driving force for swelling and spreading. Alternatively, cell division pushes microorganisms against each other or against the produced matrix, leading to a pressure buildup and expansive sliding. Such processes can be facilitated by reducing the surface tension of the biofilm through the action of surfactants secreted by the cells.

While the exact mechanisms controlling the expansion of *K. sucrofermentans* biofilms are unknown, it is reasonable to assume that the sliding-promoting mechanisms described above will be enhanced for cells displaying high growth rates. On this basis, the rate of radial expansion of biofilms is expected to increase with the rate of cell growth. Under this assumption, it is worth noting that the biofilm spreading rate of Native bacteria is comparable to that of other strains (Fig. 3e) despite the lower growth rate of this microorganism (Fig. 1c). This suggests that the Native strain must benefit from other factors impacting its radial expansion rate. Since spreading may also be affected by interactions between the biofilm and the underlying substrate,^40–42^ we infer that the Native biofilm may display lower friction with the agar gel compared to the other bacteria. Although we cannot rule out biological factors, such a biophysical explanation might be more probable in view of the high similarity of the strains used in this study.

Following this rationale, we hypothesize that the friction between the biofilm and the underlying agar substrate plays a role in the spreading behavior and extent of segregation in mixed communities. The friction defines how much the biofilm is able to expand in response to the pressure that drives spreading. This physical process is captured by the following dissipative relation: *ξu* = −∇*p*, where *ξ* is the friction coefficient between the biofilm and the substrate, *u* is the velocity field associated with biofilm expansion, and ∇*p* is the gradient in pressure that drives the process. Assuming that the film grows slowly and isotropically under viscous-dominated conditions, we expect the gradient in pressure to scale linearly with the growth rate of cells, *g*.^21,40^

Considering this scaling relation and taking the expansion rate (*u*) as the biofilm velocity in the equation above, we expect the friction coefficient *ξ*_i_ to be proportional to the ratio *g*_i_/*u*_i_ for each strain i, such that *ξ*_i_ = *k*_i_*g*_i_/*u*_i_, where *k*_i_ is the proportionality constant (Fig. 3f). In a first approximation, we assume the proportionality constants *k*_i_ to be comparable for all strains. Using this approach, *ξ*_i_ can be calculated directly from the growth and spreading experiments, thus providing a metric to quantify the level of friction experienced by the biofilms during spreading. Comparison of the *g*_i_/*u*_i_ ratios obtained from the expansion of single cultures confirms that the friction in the Native biofilm should be significantly lower than in biofilms made from the Control and Knockout strains (*P* < 0.001).

The similar spreading behavior of single cultures contrasts with the spatial patterning of cells when combined pairwise in microbial communities. This indicates that the formation of microbial patterns in two-strain biofilms cannot be directly predicted from the spreading behavior of individual strains. Instead, the observed patterns must arise from the different phenotypic traits of the investigated bacteria. Previous research has indeed shown that microorganisms can self-organize into distinct domains driven by differences in cell shape, cell growth rates, cell–cell forces, and polymer-forming ability.^22^ To quantitatively capture the differences between the three distinct patterns experimentally observed in the mixed communities, we defined a metric called the eye-score.

**Fig. 4:**
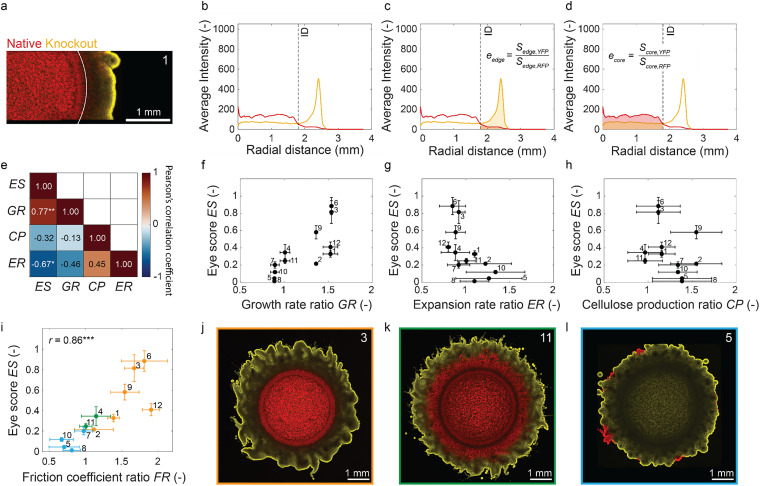
Eye-score quantification in microbial communities and relation to individual strain properties. (a) Representative confocal image of a microbial community biofilm formed by the Native and Knockout strains. Biofilms made by other strain pairs are displayed in the Fig. S4. The threshold inner diameter between the core and edge of the biofilm is shown in white. (b) Representative azimuthal average intensity of cell fluorescence as a function of the radius of the biofilm (RFP in red; YFP in yellow; others in SI, Fig. S5). The inner diameter (ID) is shown as a dashed line. (c and d) Quantification of the enrichment factor of the outer strain at the edge *e*_edge_ (c) and at the core *e*_core_ (d). The eye-score is calculated as *e*_edge_/*e*_core_ and normalized to the highest eye-score to give *ES*. (e) Pearson's linear correlation coefficients between the normalized eye-score (ES) and the property mismatch of individual strain pairs (edge strain/core strain). (f) Eye-score correlates positively with the growth rate mismatch (GR = *g*_edge_/*g*_core_). (g) Eye-score correlates negatively with the biofilm expansion rate mismatch (ER = *u*_out_/*u*_in_). (h) Eye-score does not correlate statistically significantly with the cellulose production mismatch (CP = *w*_edge_/*w*_core_). (i) Positive correlation between the eye-score and the mismatch in friction coefficient (FR ∼ *ξ*_edge_/*ξ*_core_). (j–l) Confocal images of distinct segregation patterns: bullseye (orange), co-spreading (green), and one-strain dominated (cyan). The numbers shown inside the plots correspond to the numbers used in Fig. 2b. Error bars represent the standard deviations. Statistics: *P < 0.05, **P < 0.01, ***P < 0.001, *n*_biofilm_ = 3.

The eye-score quantifies the extent of concentric segregation between two strains by measuring how much each cell type has been enriched in the edge and in the core of a bullseye pattern (Fig. 4a–d). To calculate the eye-score, we first estimate the amount of cells in the core and in the edge of the pattern by measuring the area under the fluorescence intensity profile curve within the core (*S*_core,i_) and within the edge (*S*_edge,i_) for each individual strain i (Fig. 4c and d). This is then used to calculate the enrichment factor (*e*) of the outer strain in the core and in the edge of the bullseye.

Taking hypothetical strains A and B as an example, the enrichment factor of A relative to B in the edge is *e*_edge_ = *S*_edge,A_/*S*_edge,B_. Similarly, the enrichment factor of A relative to B in the core is *e*_core_ = *S*_core,A_/*S*_core,B_. Since maximum segregation occurs when A is enriched in the edge (high *e*_edge_) but depleted in the core (low *e*_core_), the eye-score is defined as the ratio *e*_edge_/*e*_core_. Considering that the initial culture contains equal amounts of A and B (*e*_edge_ = *e*_core_ = 1), no segregation leads to an eye-score of 1. By contrast, strong segregation results in *e*_edge_ ≫ 1 and *e*_core_ ≪ 1, and therefore an eye-score much higher than 1. In order to facilitate comparisons across different microbial communities, the absolute eye-score was divided by the highest value among all measured patterns to obtain the normalized eye-score, ES.

The radial intensity profiles from the imaged mixed biofilms were used to calculate the eye-score of all the observed patterns (Fig. S4 and S5). By comparing the eye-score to the previously classified colony categories, we found that the bullseye, the one-strain dominated, and the two-strain co-spreading patterns display mean ES values predominantly within the ranges 0.21–0.88, 0.01–0.20, and 0.25–0.35, respectively. As expected, the bullseye patterns exhibited greater ES values, while the one-strain dominated patterns showed the lowest ES values (Fig. S6). This indicates that the eye-score quantitatively captures the concentric segregation observed in the mixed biofilms.

To understand the potential role of phenotypic traits in the formation of spatial patterns in mixed biofilms, we studied how differences in growth rate, cellulose production, and expansion rate affect cell segregation. The differences in individual traits were quantified by dividing the properties of the strain on the edge over those of the core strain for each pair of bacteria. The growth rate ratio is given by GR = *g*_edge_/*g*_core_, the cellulose production ratio is CP = *w*_edge_/*w*_core_, and the biofilm expansion rate ratio is defined by ER = *u*_edge_/*u*_core_. We call these ratios the mismatch in phenotypical traits. Using these property ratios, we explored experimental correlations between the spatial patterns of the mixed microbial biofilms and trait mismatches among the individual strains.

The impact of phenotypic mismatch on the segregation of the biofilms was assessed by comparing the ES of the communities with the individual trait ratios using Pearson's correlation coefficients (Fig. 4e–h). Despite the relatively low number of replicates per condition (*n* = 3), statistically meaningful correlations were obtained in this analysis. The results reveal that the ES exhibits a positive, linear correlation with the growth rate ratio GR (*r* = 0.77, *P* < 0.01) and a negative, linear correlation with the expansion rate ratio ER (*r* = −0.67, *P* < 0.05). The fact that the ES correlates positively with the growth rate (*g*) ratio and negatively with the expansion rate (*u*) ratio suggests that the friction coefficient between the biofilm and the substrate (*ξ* ∼ *g*/*u*) may also govern the spatial segregation of the mixed communities.

To assess the possible role of friction on the spatial organization of mixed communities, we compared the ES with the friction coefficient mismatch estimated for the distinct strain pairs (Fig. 4i). In this analysis, the friction coefficient mismatch between two separate strains in a biofilm (FR) was approximated by the ratio: *ξ*_edge_/*ξ*_core_ = GR/ER, assuming equal proportionality constants (*k*_i_) for all strains. Noticeably, we found that the ES displays a strong linear correlation with the friction coefficient ratio between strains of the microbial communities (*r* = 0.86, *P* < 0.001). In other words, larger mismatches in friction coefficient between strains lead to stronger cell segregation in the mixed biofilms. The observed correlation between the ES and the FR should still be observed under other biofilm spreading conditions, as long as the growth rate and/or the expansion rate of the individual strains show a linear dependence on the parameter controlling spreading. For example, if we take the nutrient concentration as the parameter controlling spreading, such linear dependence would mean that a two-fold increase in nutrient concentration would increase the growth rate and/or the expansion rate of both strains by a factor of 2, resulting in the same friction coefficient ratio (FR) and same spatial pattern (ES).

In contrast to the growth rate and the expansion rate, the mismatch in cellulose-forming ability of the microorganisms (CP) showed no significant linear correlation with the eye-score ES (Fig. 4h). Given the large standard deviation obtained for the cellulose production data, more replicates would be required in future research to identify possible correlations between pattern formation and cellulose production in these microbial communities. Importantly, the ability to overproduce cellulose is also found to be beneficial for the strain by providing access to nutrients and oxygen at the edge of the colony. Indeed, we observed that cellulose overproducers tend to occupy the edge of the biofilm, whereas the underproducers are usually found in the core of the microbial colony (Fig. 2). The only case in which the cellulose overproducer is located in the core was found in the co-spreading pattern (pairs 4 and 11). It should be noted though that the mean cellulose-forming ability of the two strains in those pairs is almost equal. Such finding agrees with previous simulations and experimental research on soil bacteria, which showed that the secretion of polymers pushes cells to the edge of the colony, leaving non-polymer producers in the core of the film.^22,43–45^ In analogy to plant cells seeking light, bacteria seem to have evolved polymer-forming abilities to reach the higher oxygen concentrations at the edge of biofilms, thereby preventing competitors from accessing this essential molecule.^44^

Taken together, our analysis suggests that the unique bullseye pattern of communities with high eye-score (ES > 0.21–0.88, orange, Fig. 4j and Fig. S7) emerges from the outward motion of the dominant cellulose overproducers at the colony edge combined with the low-friction underproducing strain remaining at the core of the biofilm. For the co-spreading pattern (0.25 < ES < 0.35, green, Fig. 4k), the comparable cellulose-forming ability and friction coefficient of the two strains lead to competitive outer growth with the lower-friction strain occupying the colony core. Finally, the one-strain dominated pattern (ES ≤ 0.20, cyan, Fig. 4l) arises when the underproducing strain features a high friction coefficient and is outgrown by the cellulose overproducer throughout the entire microbial community.

In some of the one-strain dominated patterns, we observe domains of the less-abundant cellulose underproducers at the edge of the biofilms. The mechanisms that allow those isolated underproducer domains to reach the edge remain unclear. These isolated cells may be free riders that position themselves at rare locations on the edge of the initial inoculum and profit from the high cellulose production of the dominating strain during biofilm expansion. Further time-resolved studies are required in future research to elucidate the mechanisms underlying this interesting experimental observation.

**Fig. 5:**
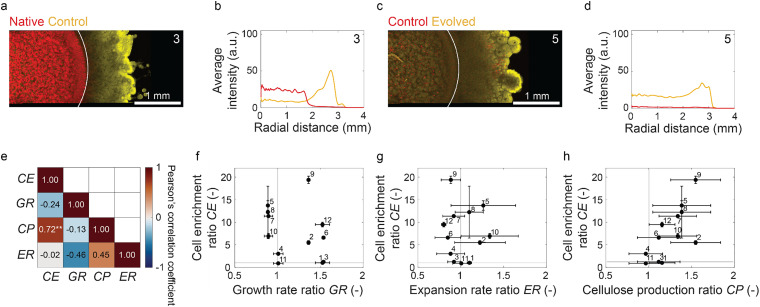
Cell enrichment in microbial community biofilms. (a and c) Representative summed *z*-stacks and (b and d) associated radial intensity profiles obtained from confocal images of two exemplary biofilms. From an initial cell ratio of 1 : 1, the final ratio of the two strains in the biofilm was 1 : 1 in (a and b) and 14 : 1 in (c and d). (e) Pearson's linear correlation coefficients between cell enrichment factor (CE = *a*_edge_/*a*_core_), and mismatch in growth rate (GR = *g*_edge_/*g*_core_), cellulose production ability (CP = *w*_edge_/*w*_core_), and radial expansion rate (ER = *u*_edge_/*u*_core_). (f–h) Cell enrichment factor of the different biofilms *versus* the mismatch in traits of the individual strains. Error bars represent the standard deviations. Statistics: **P < 0.01, *n*_biofilm_ = 3.

Beyond spatial patterns, phenotypic traits of individual strains are also expected to affect the cell composition in the grown two-strain biofilms. To investigate this, we quantified the amount of cells of each strain i (*a*_i_) from the individual fluorescent channels of the microbial communities images (Fig. S8). From these experiments, a cell enrichment factor was calculated: CE = *a*_edge_/*a*_core_. The resulting cell enrichment factors show that the cell composition of the final biofilm was either kept close to the initial 1 : 1 inoculum ratio (Fig. 5a and b) or was dominated by one strain (Fig. 5c and d).

The enrichment of a microbial community with one cell type suggests that one or more phenotypic traits of the dominating microorganism allow it to outcompete the other strain present in the colony. To identify possible traits responsible for such a competitive advantage, we compared the measured cell enrichment factor (CE) with the mismatch in cell growth rate (GR), cellulose-forming ability (CP), and expansion rate (ER) between the individual strains (Fig. 5e–h). The influence of such individual traits on cell enrichment was quantified using Pearson's correlation coefficients (Fig. 5e).

The experimental data reveal a statistically significant linear correlation between the cell enrichment factor and the mismatch in cellulose-forming ability of the constituent bacterial strains (*r* = 0.72, *P* < 0.01) (Fig. 5h). This means that biofilms containing strains with similar cellulose-forming abilities could maintain their cell ratio closer to the initial 1 : 1 inoculum, whereas larger mismatches in cellulose production gave an advantage to the strain able to produce more polymer fibers. In contrast to the eye-score, the cell growth rate and expansion rate mismatches were found to correlate weakly with the measured cell enrichment factor (Fig. 5f and g). Overall, our experiments reveal that cellulose overproduction is an advantageous trait that enables bacteria to dominate the cell population and occupy a nutrient-rich position in the microbial community.

## Materials & methods

### Bacterial strains

The four strains studied here are *Komagataeibacter sucrofermentans*, known to produce cellulose extracellularly. The Native strain (JCM 9730) was obtained from the American Type Culture Collection (ATCC 700178). The other strains were the result of a previous study.^39^ The Control strain is genetically identical to the Native strain but was stressed with external factors, namely lack of nutrients and light for a period of time. The Evolved strain (JML 2321) is the result of a directed evolution process aimed at increasing bacterial cellulose production of the Native strain. Compared to the Native strain, the Evolved strain has a 12-base pair deletion in *clpA* at the binding site with *clpS*, likely disturbing the ClpA–ClpS interaction. The Knockout strain (JML KO23) was engineered from the Native strain to feature a missing *clpS* start codon (ΔclpS::cat) that entirely suppresses the ClpA–ClpS interaction. ClpAPS is a protease complex that participates in protein turnover in cells. All four strains were grown using a medium composed of 25 g L^−1^d-mannitol (Thermo Fisher Scientific), 5 g L^−1^ yeast extract (Sigma-Aldrich), 3 g L^−1^ peptone (Sigma-Aldrich), and 15 g L^−1^ agar (Sigma-Aldrich) for solid medium.

### Growth rate estimation

Growth curves were measured for each of the four strains in triplicates. Frozen stocks (−80 °C) were thawed, spun down (3000 rcf, 10 min), and resuspended in fresh medium. After adjusting their absorbance at 600 nm to 0.005 (∼1.5 × 10^6^ CFU mL^−1^, Varioscan LUX, Thermo Fisher Scientific), 2 vol% of sterile-filtered cellulase solution (*Trichoderma re*es*ei* ATCC 26921, Sigma-Aldrich) was added to prevent the formation of a cellulose pellicle. Absorbance measurements were recorded at 600 nm in 24-well plates (flat bottom, Techno Plastic Products, TPP), each well containing 1 mL culture. The well plates were covered with a breathable film (BREATHseal^TM^, Greiner Bio-One) to avoid cross-well contamination and incubated at 28 °C and 200 rpm under 80% relative humidity (RH). Every day, the film was taken off for measurements and replaced by a new one. Blanks composed of media with 2 vol% cellulase solution were subtracted from the data points. Each growth curve was fitted with the following logistic growth model: 
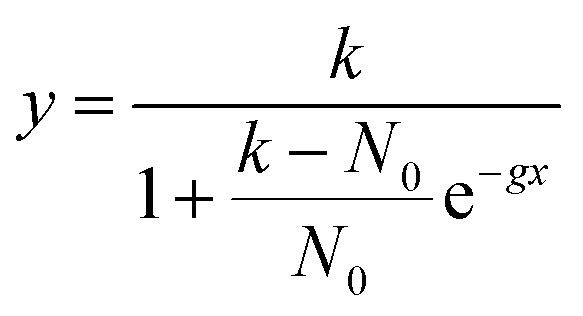
 where *y* is the measured absorbance of the cell suspension, *x* is the time, *k* is the carrying capacity, *N*_0_ is the initial population size, and *g* is the growth rate. Considering *N*_0_ as the initial absorbance measurement, *k*_i_ and *g*_i_ could be estimated for each growth curve (i = {Native, Control, Evolved, Knockout}).

### Production of cellulose pellicles

Frozen stocks (−80 °C) from all four strains were thawed, streaked onto solid medium, and incubated at 28 °C under static conditions for 7 days (80% RH) to obtain single colonies. The colonies were then inoculated in 5 mL liquid medium in 50 mL Falcon tubes (TPP) closed with a foam plug. Nine replicates were produced for each strain. After 12 days of incubation at 28 °C (static, 80% RH), the cellulose pellicles formed at the air-medium interface were retrieved and washed three times in 50 mL of distilled water (MilliQ). Washed pellicles were then put for 1 hour in 50 mL of 0.1 M NaOH solution (Fisher Scientific) at room temperature and finally brought back to neutral pH with additional MilliQ water washes. The air-dried pellicles were weighted using a precision balance (UMT2 Microbalance, Mettler Toledo) to determine the amount of cellulose produced on average by each strain *w*_i_ (i = {Native, Control, Evolved, Knockout}).

### Genetic engineering

Each of the four strains was engineered through chromosomal insertion of either a red or a yellow fluorescent protein. This was achieved through homologous recombination using plasmid vectors bearing an insertion cassette consisting of a kanamycin resistance gene (*kanR*) and either *mCherry* or *mVenus* fluorescent proteins driven by the strong constitutive promoter J23104. pUC19 was used as the plasmid backbone, as it is unable to replicate within *K. sucrofermentans*. The insertion cassette was flanked by 500 bp regions of homology to the genome, labeled as left (L) and right (R) regions.

Cloning was performed using Gibson Assembly^46^ of PCR fragments amplified using Q5 polymerase (NEB). PCR fragments were isolated by gel electrophoresis and cleaned using Wizard SV Gel and PCR Clean-Up System (Promega). Cloning and plasmid amplification was performed in *E. coli* DH5a grown with 100 μg mL^−1^ Carbenicillin, and plasmids were isolated by miniprep using the PureYield Plasmid Miniprep System (Promega).

#### Location determination

Three genomic insertion sites were initially chosen in intergenic regions, as we aimed to minimally disrupt native gene expression patterns. The three sites were located on the *K. sucrofermentans* DSM 15973 genome (NCBI Accession CP137157.1) at genomic positions 803 100, labeled site 8 (s8), 1 080 200 (s11), and 2 681 520 (s26). 500 bp regions around the insertion site were amplified by PCR using the primers shown in Table S1, and these were used as homology regions for recombination.

Six plasmids were built in total, the YFP bearing pUCKO-KY-s8, pUCKO-KY-s11, pUCKO-KY-s26, and the RFP-bearing pUCKO-KR-s8, pUCKO-KR-s11, pUCKO-KR-s26. Homology regions and insertion cassettes were verified by Sanger sequencing by Microsynth (Switzerland). pUCKO-KY-s26 and pUCKO-KR-s26 sequences and physical DNA are available from AddGene.

Of the three insertion sites, s11 consistently showed poor transformation efficiency and was not characterized further. The remaining insertion sites, s8 and s26, were tested in the Native strain with the RFP inserts for fluorescence. s26 showed consistently higher fluorescence values (Fig. S9), and thus was selected for further use.

#### Transformation

To prepare electrocompetent *K. sucrofermentans* cells, 300 mL cultures were grown with 2 vol% cellulase solution until the exponential phase. The cells were then centrifuged (300 rcf, 10 min, 4 °C) and resuspended in 10% glycerol solution at 4 °C three times, each time reducing the volume (300 mL, 150 mL, and 5 mL). The resulting electrocompetent cells were aliquoted in 4 °C Eppendorf tubes, frozen with liquid nitrogen, and stored at −80 °C until further use.

For genomic insertion, 100 μL aliquots of electrocompetent *K. sucrofermentans* cells were transformed with the pUCKO plasmids using the MicroPulser Electroporator (BioRad) and 0.2 cm gap cuvettes, using the Ec2 setting. Electroporated cells were suspended in 1 mL of sterile growth media containing 2 vol% cellulase and left to recover overnight at 28 °C in a shaking incubator. In the following day, 200 μL of each recovery culture was plated onto an agar plate containing 10 μg mL^−1^ kanamycin and grown statically at 28 °C for 5 days. Correct insertions were verified by PCR using primers around the insertion site (*i.e.* primer s26_LF and s26_RR for s26), which in each case yielded fragments with lengths that incorporated the insertion cassette. Transformed *K. sucrofermentans* colonies were also tested for pUCKO plasmid presence by plating onto plates with 100 μg mL^−1^ carbenicillin, and none were found to be resistant.

### Production of cellulose biofilms

Before solidifying, 1 mL of solid medium was added to each well of 12-well plates (Techno Plastic Products AG). Once the medium solidified, a 1 μL drop of concentrated cell suspension (44 × 10^6^ CFU mL^−1^) was deposited at the center of each well. The cell suspension either contained a single strain or a 1 : 1 mixture of RFP- and YFP- expressing strains. The well plates were finally parafilmed and incubated for 11 days at 28 °C under static conditions and 80% RH. For biofilms produced by single strains, 218 μM of Fluorescent Brightener 28 (Sigma-Aldrich) was added to the medium after autoclaving to stain the produced cellulose.

### Confocal microscopy

Confocal microscopy was used to image the biofilms directly on the solid medium (TCS SP8, Leica) in 12-well plates (TPP). Using a 10× objective (0.32 NA, HC PL FLUOTAR), *z*-stacks were taken for each biofilm (30 slices, 10 μm spacing), over the whole biofilm area for one replicate, and over a representative section for the other replicates to reduce imaging time (512 × 512 resolution). YFP- and RFP-expressing bacteria were imaged simultaneously. YFP-expressing bacteria were excited with a 514 nm laser and detected between 520 nm and 540 nm, whereas RFP-expressing bacteria were excited with a 552 nm laser and detected between 587 nm and 638 nm. When stained, the cellulose was imaged sequentially using a 405 nm laser with the emission set between 430 nm and 460 nm to avoid signal overlapping.

### Image analysis

All the image processing was performed using ImageJ.^47^ First, confocal *z*-stacks were summed, merged using the Grid/Collection stitching plugin,^48^ and resized to the biggest image's dimensions.

#### Single-strain biofilms for radial expansion rate

Cellulose-stained biofilms produced by single strains either expressing RFP or YFP were imaged daily for 11 days (Fig. S1). The Radial Profile plugin^49^ was used to extract the azimuthally integrated intensity of the cellulose fluorescence channel for the images of full biofilms. For the biofilms imaged only on a representative portion, the Plot Profile function from ImageJ^47^ was used instead. The fluorescence intensity profiles were imported into MATLAB (R2023a, MathWorks), where the image analysis was performed. Biofilm diameters were defined as the distance at which the maximum intensity decreases by half in the profiles, starting from the edges of the biofilms. The maximal expansion rate 
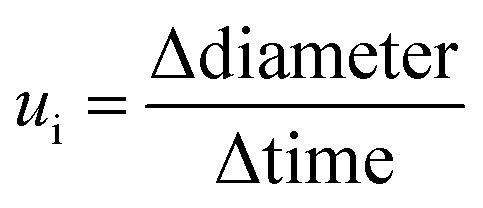
 of the biofilms was then calculated between day 2 and day 5 for each strain i (i = {Native_RFP_, Native_YFP_, Control_RFP_, Control_YFP_, Evolved_RFP_, Evolved_YFP_, Knockout_RFP_, Knockout_YFP_}).

#### Microbial community biofilms for eye-score

Biofilms produced by strain pairs were imaged after 11 days of incubation. The YFP signal was found to bleed through the RFP channel, thus requiring a correction. To do so, biofilms containing only YFP-expressing cells were imaged with both the YFP and RFP channels (Fig. S10). A bleed-through factor was defined as 
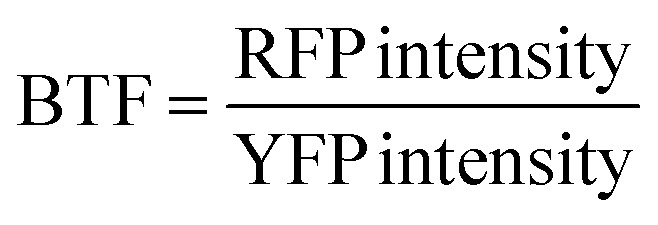
. The RFP channel of all images was then corrected as follows: RFP_corrected_ = RFP_original_ − (BTF × YFP_original_). Quantitative image analysis was conducted on the images of biofilm sections. The Radial Profile Angle plugin^50^ was used to extract the azimuthally integrated intensity of the different fluorescence channels over a 12° angle to match the images’ height. The RFP and YFP fluorescence radial intensity profiles were imported into MATLAB (R2023a, MathWorks), where the image analysis was performed.

### Microbial community properties

#### Eye-score determination

First, an inner diameter (ID) had to be determined for each image to separate the core and the edge of the biofilms. To do so, the RFP and YFP radial intensity profiles were smoothed over 45 points. Using the profile corresponding to the outer strain and starting from the outside, the ID was defined as the radius (*r*) at which a local minimum in the fluorescence intensity was found. This position was determined by finding the radius for which the first derivative of the intensity is equal to zero and the second derivative of the intensity is positive (Fig. S5). The obtained IDs were drawn on the confocal images and manually adjusted when necessary (Fig. S4). The amount of cells from each strain i at the edge and in the core was estimated by measuring the area under the corresponding fluorescence intensity profiles at the edge (*S*_edge,i_ for *r* > ID) or within the core (*S*_core,i_ for *r* < ID). This was then used to calculate the enrichment factor (*e*) of the outer strain A relative to the inner strain B, both at the edge and the core. At the edge, the enrichment factor was *e*_edge_ = *S*_edge,A_/*S*_edge,B_, while in the core, it was *e*_core_ = *S*_core,A_/*S*_core,B_. The eye-score was finally defined as *e*_edge_/*e*_core_ and normalized by the highest obtained eye-score. The normalized eye-score is referred to as ES.

#### Cell enrichment factor

For each microbial community, the RFP- and YFP-expressing cell amounts *a*_i_ were measured from the radial intensity profiles of each strain i as the area under the entire curve in arbitrary units. The overall cell enrichment ratio was calculated as the total cell amount of the strain on the edge over the core strain cell amount: 
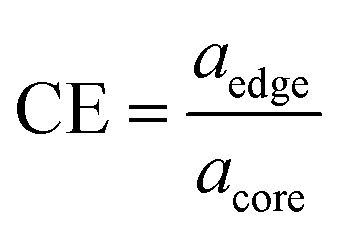
.

#### Ratios of single-strain properties

Ratios of properties between strain pairs in microbial community biofilms were calculated by dividing the property of the strain on the edge and the property of the core strain. The growth rate ratio was given by 
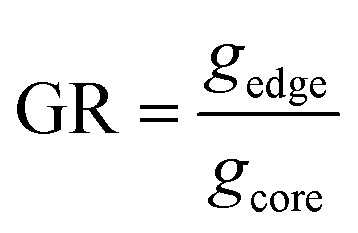
, the cellulose production ratio was taken as 
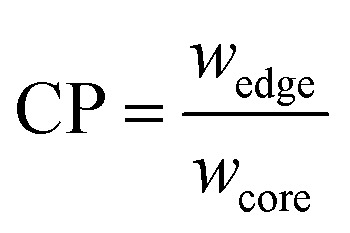
, and the biofilm expansion rate ratio was calculated from 
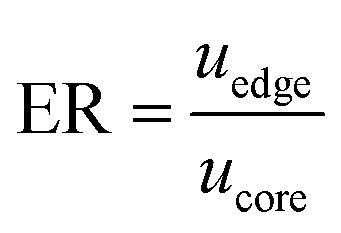
.

### Statistics

All statistical analyses were performed using MATLAB R2023a (MathWorks). To compare the properties of the individual strains (Native, Control, Evolved, Knockout), one-way ANOVAs were conducted. Alternatively, two-way ANOVAs were conducted to compare strains (Native, Control, Evolved, Knockout) and fluorescent protein expression (RFP, YFP). If the result showed a statistically significant difference between the groups (*P* < 0.05), pairwise comparisons were used with a Bonferroni correction to compensate for the effects of multiple comparisons. Linear Pearson's correlation coefficients were calculated between the mean properties of biofilms produced by microbial communities.

## Conclusions

Microbial communities of cellulose-producing bacteria can form exquisite spatial patterns during biofilm spreading on solid culture medium. The biofilm patterns emerge when *K. sucrofermentans* species carrying simple mutations or previously subjected to environmental stresses are combined pairwise. Spatial patterns result from the self-organization of cells into segregated domains depending on the pair of bacteria present in the initial inoculum. Notably, cell segregation occurs even for genetically identical strains. The level of concentric cell segregation was found to correlate linearly with the mismatch in friction between each cell type and the underlying substrate. Cell pairs displaying friction mismatches lead to highly segregated domains, with the lower-friction cell type in the core and the higher cellulose producer at the edge of an eye-like pattern. Conversely, biofilms containing a core strain with a higher friction coefficient become dominated by the bacteria that produces a larger amount of cellulose. Cell co-spreading occurs when both bacterial strains show similar friction and cellulose-forming capabilities. Moreover, the ability of bacteria to overproduce cellulose allows them to position themselves at the nutrient-rich edge of the biofilm and overgrow the other variant during film spreading. Tuning the cellulose-forming ability and friction of bacterial biofilms provides an attractive route to control cell composition and self-organization in microbial communities of relevance in health, materials fabrication, and biotechnologies.

## Author contributions

J. M. L. and A. R. S. conceptualized the project. A. K., M. S., and A. R. S. supervised the project. J. M. L., A. K., M. S., and A. R. S. participated in experimental design. J. M. L. performed all experiments, including growth curves, cellulose production, biofilm spreading, confocal imaging, and image analysis. A. K. genetically engineered the different bacterial strains to express fluorescent proteins, with the support of J. M. L. J. M. L. and A. R. S. drafted the manuscript and the figures. A. R. S. and J. M. L. wrote the manuscript. J. M. L., A. K., M. S., and A. R. S. discussed the results and contributed to the final version of the manuscript.

## Conflicts of interest

The authors have filed a patent application related to the evolved bacteria utilized in this work.

## Supplementary Material

SM-021-D5SM00720H-s001

## Data Availability

The data supporting this article have been included as part of the supplementary information (SI). Supplementary information is available. See DOI: https://doi.org/10.1039/d5sm00720h.
