# Supplementary material for: Self-organization of cellulose-producing microbial communities during biofilm spreading
Source: Soft Matter. 2025 Oct 31;21(44):8543–54. doi: 10.1039/d5sm00720h (PMC12576783; doi:10.1039/d5sm00720h)
Supplement: SM-021-D5SM00720H-s001 [file SM-021-D5SM00720H-s001.pdf]

Supplementary Information (SI) for Soft Matter.
This journal is © The Royal Society of Chemistry 2025

Supplementary Information

Self-organization of cellulose-producing microbial communities during biofilm spreading

Julie M. Laurent, Anton Kan, Mathias Steinacher, André R. Studart*

Complex Materials, Department of Materials, ETH Zürich, 8093 Zürich, Switzerland

* Corresponding author: andre.studart@mat.ethz.ch

Content: Table S1 & Figures S1-S9

Tables

Table S1. Primers used to amplify homology regions for genomic insertion. Capitalized DNA is the homologous to the *K. sucrofermentans* genome and the rest of the primer contains an overlap region for Gibson Assembly.

S8_LF	tacaacgtcgtgactgggaaaaccctggcgATAGGCGTAGCTGACATCATGC
S11_LF	tacaacgtcgtgactgggaaaaccctggcgTTGTAATCTACCATATAAGCAACCG
S26_LF	tacaacgtcgtgactgggaaaaccctggcgATTGCGCGCGCGGATCAGG
S8_LR	accttgcccgtttttttgccggagctagtaAATCCGGGGAAGCCGCCC
S11_LR	accttgcccgtttttttgccggagctagtaAGGTCGGGAGACCTGTTTAC
S26_LR	accttgcccgtttttttgccggagctagtaGCCCCGCGTACTGGCCGATTC
S8_RF	ctatcaacaggagtccaagctgcagcggccGTAGGAAATGCATTCCTGCC
S11_RF	ctatcaacaggagtccaagctgcagcggccGCTTCAGGATGTCTGCCTTTTATTC
S26_RF	ctatcaacaggagtccaagctgcagcggccCTGCGGGGCGTTCCACCAC
S8_RR	gatgtgctgcaaggcgattaagttgggtaaTTCCCCGGTCCCTGCGTC
S11_RR	gatgtgctgcaaggcgattaagttgggtaaGCCCAGCGAAGTATAGGCG
S26_RR	gatgtgctgcaaggcgattaagttgggtaaCTGCGCGTGCGGCTGAAAG

Figures

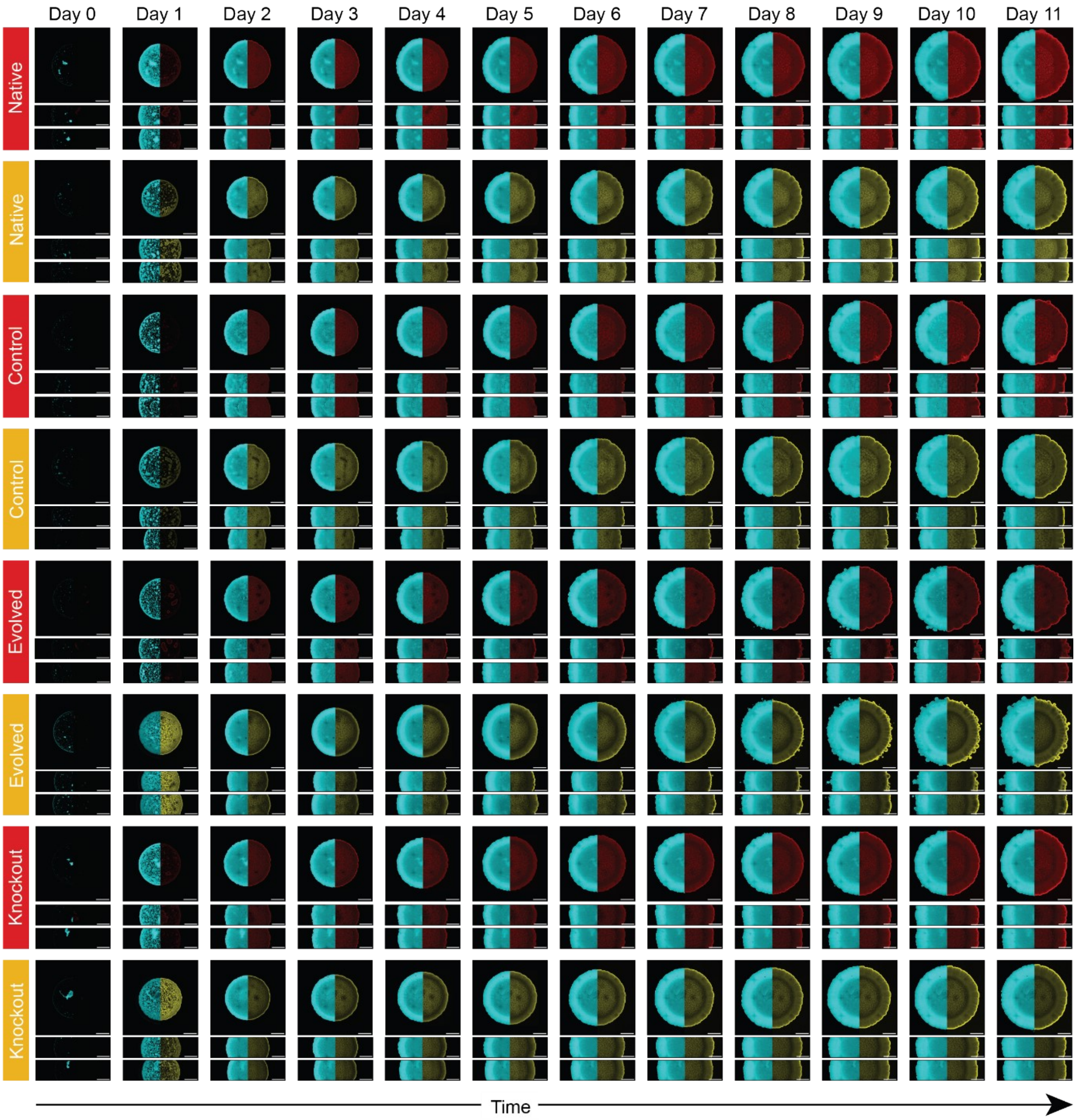

Figure S1. Confocal images of cellulose biofilms grown from individual strains over 11 days. The cell cultures were deposited on top of 1.5% agar medium containing a cellulose-binding fluorescent dye (cyan, left part of images). The strains expressed either RFP or YFP (red or yellow, right part of images). Triplicates were made for each strain to extract the diameters of the biofilms over time using the cellulose fluorescence. Scale bars: 1 mm.

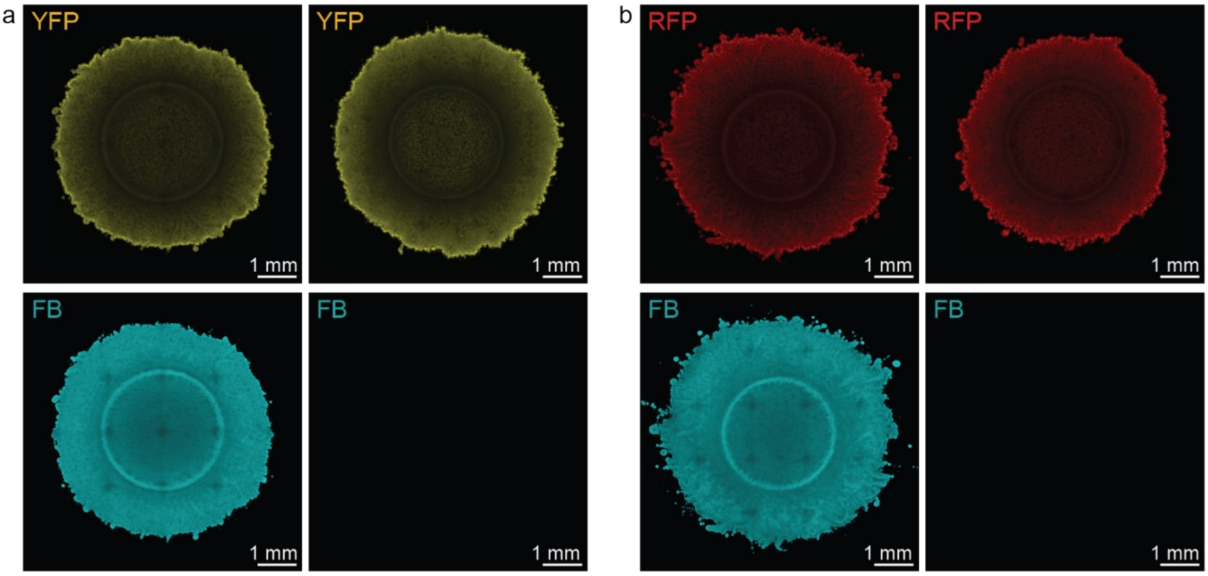

Figure S2. Effect of the cellulose-binding dye added to the solid medium on the spreading of single-strain biofilms. (a,b) Confocal images of biofilms produced by (a) YPF-expressing or (b) RFP-expressing Native strain taken after 17 days of incubation on agar medium. The images on the left of (a) and (b) were obtained from biofilms containing the cellulose-binding dye Fluorescent Brightener 28 (FB), whereas no dye was present in the biofilms shown on the right. Visually, no striking difference was observed between the distinct biofilms.

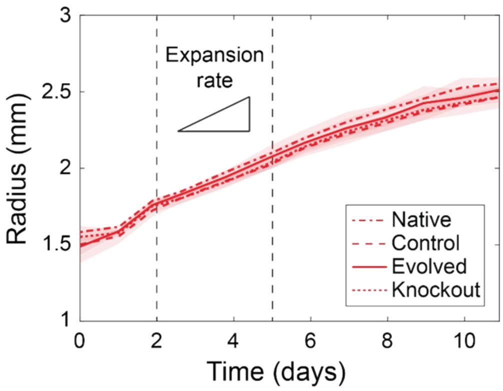

Figure S3. Radius of biofilms made by single strains over time extracted from the integrated cellulose-fluorescence curves for all RFP-expressing strains. The maximal expansion rate is determined between Day 2 and Day 5 (dashed lines). Shaded areas around the curves represent the standard deviations.

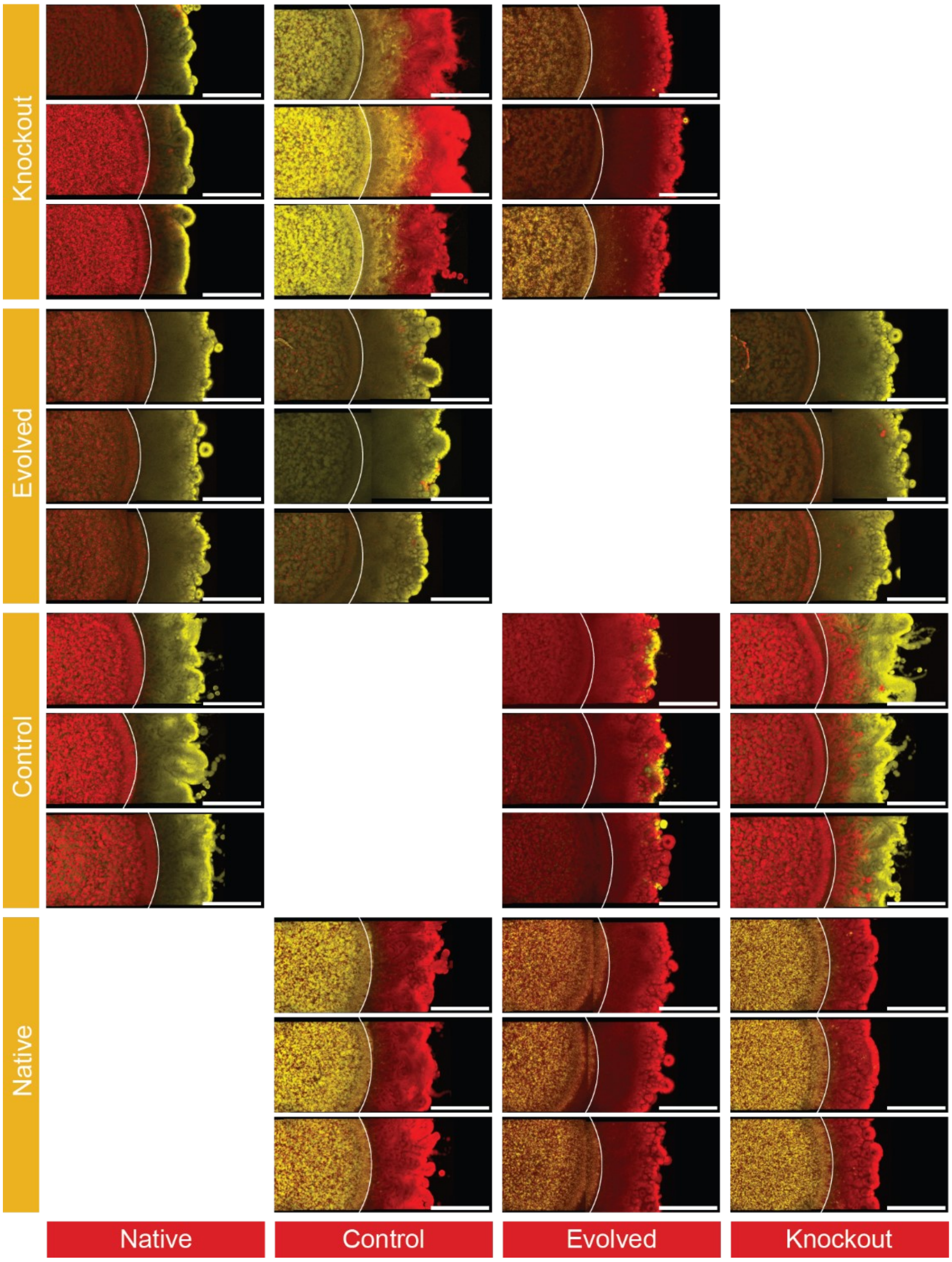

Figure S4. Confocal images of microbial communities used for the eye-score quantification. RFP- and YFP-expressing bacterial strains were mixed in 1:1 ratio before deposition on top of 1.5% agar solid medium and imaged after 11 days of incubation ($n_{biofilm}$ = 3). The inner diameter used as a threshold between the edge and the core of the biofilms for the eye-score calculation is shown in white. Scale bars: 1 mm.

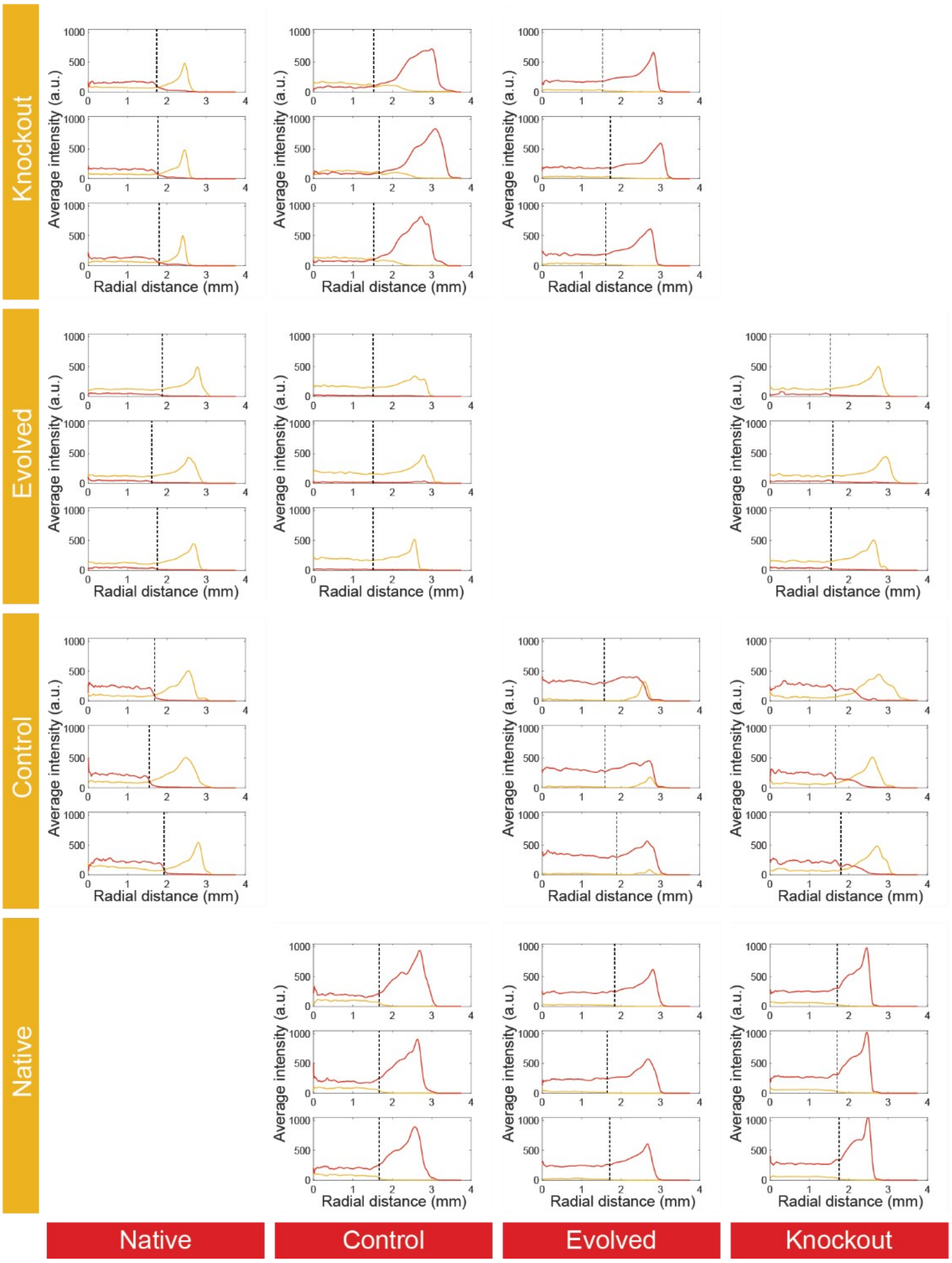

Figure S5. Azimuthal average intensity profiles of RFP- and YFP-expressing cells extracted from each confocal image of microbial communities. The dashed line on each graph represents the inner diameter used as a threshold between the edge and core of the biofilms for the eye-score calculation. $n_{biofilm}$ = 3.

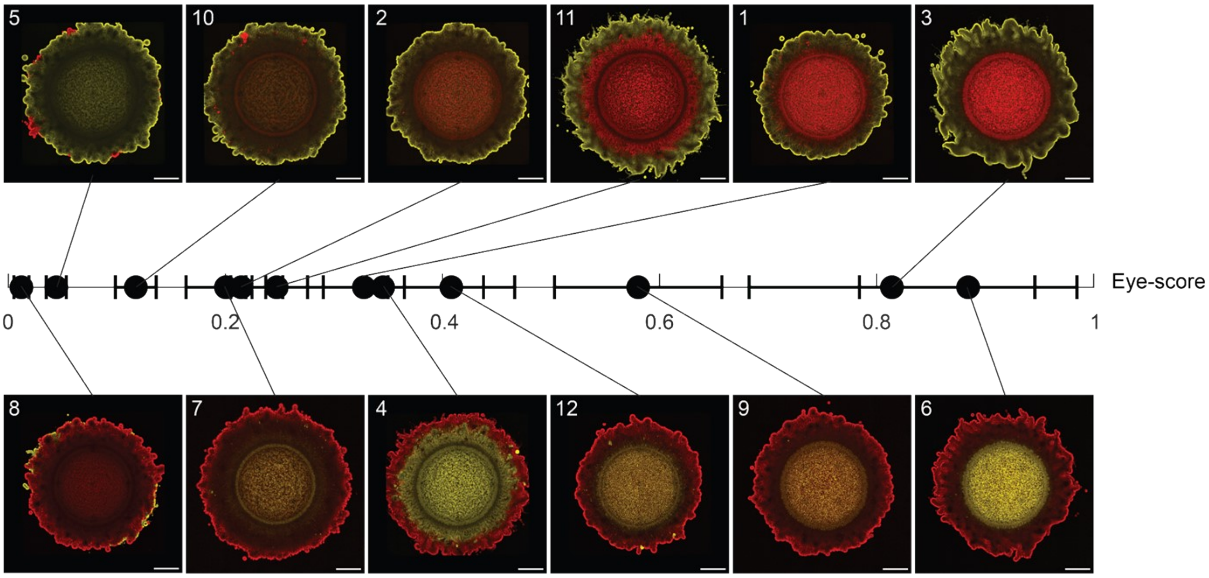

Figure S6. Confocal images of mixed communities displayed along an eye-score scale. The score is calculated for each pair of strains as the enrichment of the outer strain at the edge (e_{edge}) over the same enrichment at the core (e_{core}). The score values obtained for each biofilm are normalized to the highest score to achieve *ES* values varying from 0 to 1. The higher the eye-score, the more concentrically segregated the bacterial strains are. The numbers shown inside the images correspond to the pair of strain numbers used in Figure 2b (main text). Error bars represent the standard deviation, $n_{biofilm}$ = 3. Scale bars: 1 mm.

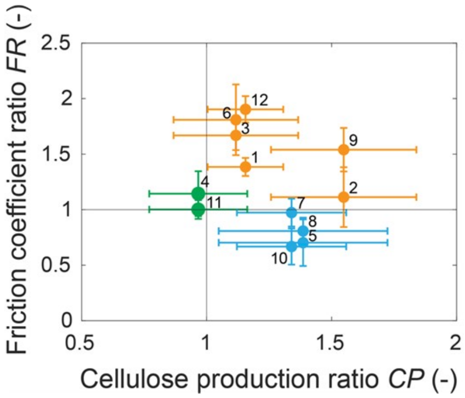

Figure S7. Selected phenotypic trait ratios of mixed communities with distinct spatial patterns. The microbial communities were mapped according to the friction coefficient ratio and the cellulose production ratio of the constituent cells. Bullseye, one-strain dominated, and two-strain co-spreading patterns are displayed in orange, cyan, and green, respectively. The numbers shown inside the plots correspond to the pair of strain numbers used in Figure 2b (main text).

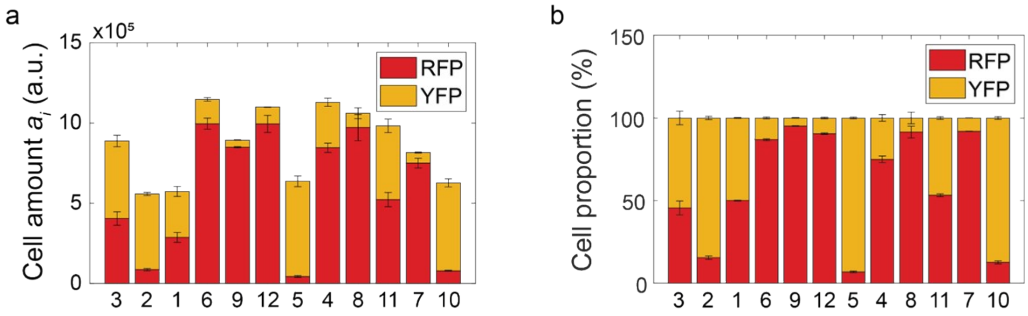

Figure S8. Relative amounts of cells expressing RFP and YFP in the microbial communities. The area under the azimuthal averaged intensity curves (a_i) were used as a measure of the cell amount on each confocal image. **(a)** Absolute a_i values for each strain i = {$Native_{RFP}$, $Native_{YFP}$, $Control_{RFP}$, $Control_{YFP}$, $Evolved_{RFP}$, $Evolved_{YFP}$, $Knockout_{RFP}$, $Knockout_{YFP}$}. **(b)** Proportion of cells in each mixed community estimated from the absolute a_i values. The numbers shown along the x-axis correspond to the pair of strain numbers used in Figure 2b (main text). Error bars represent the standard deviation, $n_{biofilm}$ = 3.

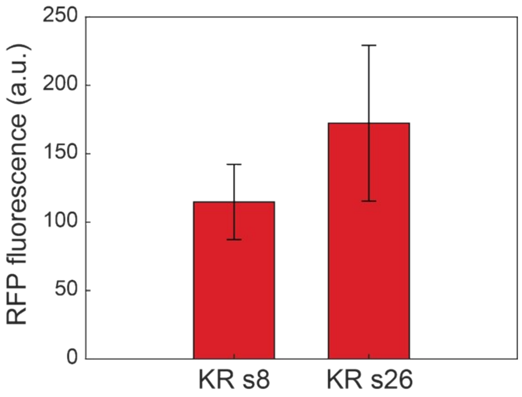

Figure S9. Insertion site selection. The Native strain was transformed with plasmids pUCKO-KR-s8 and pUCKO-KR-s26 to create stable chromosomal insertions of constitutive RFP expression. Pellicles of each strain were grown in 6-well plates and the RFP fluorescence was imaged in a ChemiDoc MP imager (Bio-Rad) with Epi-green excitation (520−545 nm) and a 650 nm emission filter. Pixel values corresponding to each pellicle were averaged. Experiment was performed in duplicate.

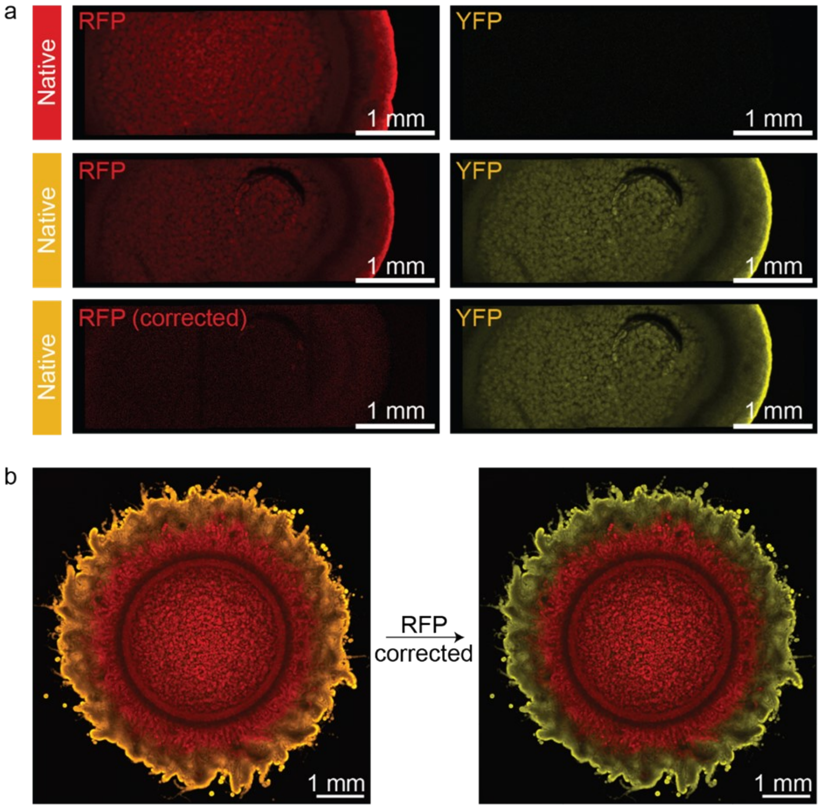

Figure S10. Fluorescence correction of confocal images to circumvent bleeding of the YFP signal. (a) The YFP-expressing cells are detectable both on the RFP and YFP channels. A Bleed-through factor (*BTF*) was calculated for all YFP-expressing cells as *BTF = RFP/YFP*. The RFP images are then corrected as follows: $RFP_{corrected} = RFP_{original} - (BTF * YPF_{original})$. **(b)** Representative confocal image from a microbial community biofilm before and after the correction.
